# The p.Phe174Ser mutation is associated with mild forms of Smith Lemli Opitz Syndrome

**DOI:** 10.1186/s12881-016-0287-1

**Published:** 2016-03-11

**Authors:** Arianna Tucci, Luisa Ronzoni, Carlo Arduino, Paola Salmin, Susanna Esposito, Donatella Milani

**Affiliations:** Pediatric Highly Intensive Care Unit, Department of Pathophysiology and Transplantation, Università degli Studi di Milano,Fondazione IRCCS Ca’ Granda Ospedale Maggiore Policlinico, Via Commenda 9, 20122 Milan, Italy; S.C.D.U. Genetica Medica, A.O. Città della Salute e della Scienza, Torino, Italy

**Keywords:** Smith Lemli Opitz syndrome, *DHCR7*, Genotype-phenotype correlation, Cholesterol metabolism, Congenital malformation syndrome, 7-dehydrocholesterol reductase

## Abstract

**Background:**

Smith Lemli Opitz syndrome (SLOS; OMIM #270400) is an autosomal recessive metabolic disorder caused by mutations in the *DHCR7* gene. SLOS is characterized by a plethora of abnormalities involving mainly the brain and the genitalia but also the cardiac, skeletal and gastroenteric system, typical dysmorphic facial features, and variable degrees of developmental delay and intellectual disability (ID). SLOS has a broad phenotypic spectrum, ranging from multiple congenital malformation syndrome, to mild developmental delay and minor malformations. A large number of mutations have been described in the *DHCR7* gene, with few common mutations accounting for the majority of mutated alleles found in patients and a large number of very rare or even private variants. Due to the wide variety of clinical presentations, diagnosis can be difficult, especially in the milder forms of the disorder. Furthermore, establishing a molecular diagnosis can be complicated by finding variants of unknown clinical significance in such cases.

**Case presentation:**

We report a case of SLOS at the mild end of the clinical spectrum, presenting with bilateral pelvis ectasia, mild dysmorphic features and mild intellectual disability. The case is compound heterozygous for a known pathogenic mutation (c.724C > T, p.Arg242Cys) and a mutation that has only been reported once in a Portuguese patient (c.521 T > C, p.Phe174Ser) whose pathogenicity has not been yet assessed. We compared the two patients carrying the p.Phe174Ser variant and concluded that this variant is associated with mild forms of SLOS.

**Conclusion:**

We report a patient with a mild case of SLOS, highlighting the importance of recognizing subtle anomalies of the genitourinary system, associated with mild dysmorphic features and mild intellectual disability in establishing the diagnosis of mild forms of SLOS. With this report, we confirm the pathogenicity of the p.Phe174Ser variant and we also provide evidence of its association with mild forms of SLOS. This finding further facilitates the establishment of a genotype–phenotype correlation for SLOS. This helps in counselling for this disorder and in predicting therapeutic responses.

## Background

Smith Lemli Opitz syndrome (SLOS; OMIM #270400) is an autosomal recessive multiple congenital anomaly syndrome caused by mutations in the *DHCR7* gene [1–3]. The prevalence of SLOS has been estimated to range between 1:15,000 and 1:60,000 in populations of European origin [4, 5], with a carrier frequency that may be as high as 2 % in certain populations [6], making it one of the most commonly inherited metabolic defects in the Caucasian population.

SLOS has a broad phenotypic spectrum ranging from multiple congenital malformation syndrome to mild developmental delay and minor malformations [7]. Whilst individuals with milder forms of SLOS may have only subtle facial characteristics and mild to no ID, individuals affected by the most severe forms of SLOS may have life-threatening multiple congenital malformations involving the brain, heart, lungs, and genitourinary system. Structural anomalies of the brain and microcephaly along with anomalies of the genitalia (namely hypospadia) are commonly found in individuals with SLOS and help guide the diagnosis. The classical facial appearance is characterized by bitemporal narrowing, a short upturned nose with anteverted nares, epicanthus, ptosis, retrognathia, and low set ears. Y-shaped syndactyly of the second and third toes and cleft palate are common but not universal findings that help to guide the diagnosis [8].

The *DHCR7* gene encodes 7-dehydrocholesterol reductase, the ultimate enzyme of sterol biosynthesis that converts 7-dehydrocholesterol to cholesterol [9]. Patients have elevated plasma concentrations of 7-dehydrocholesterol, and most also have low circulating levels of cholesterol [4] The *DHCR7* gene encodes a protein of 475 amino acid residues. The DHCR7 protein consists of nine transmembrane (TM) domains and corresponding cytoplasmic loops of which the fourth is thought to contain the putative active site. To date, approximately 140 gene mutations have been identified across the *DHCR7* gene (http://www.hgmd.cf.ac.uk/ac/index.php 19/08/2015). The 13 most frequent mutations account for approximately two-thirds of all mutant alleles found in *DHCR7*, indicating a large number of very rare or even private mutations [10].

We report a case of SLOS at the mild end of the clinical spectrum, who is compound heterozygous for a known pathogenic mutation (c.724C > T, p.Arg242Cys) and a mutation that has only been reported once in the Portuguese population (c.521 T > C, p.Phe174Ser) [11].

## Case presentation

The proband, a 7 year old boy, was the second child of unrelated parents. His father was Italian, and his mother was of Cuban ancestry. Both his parents and his older sister were healthy. In the family history, a female cousin of his mother was reported to be mildly intellectually disabled. A male cousin of his father also had an ID.

During the pregnancy, a potential miscarriage was reported. The foetal ultrasound scans at 12 and 20 weeks of gestation reported normal growth and development. He was delivered at term by vaginal delivery. He was noted to be mildly microcephalic (occipitofrontal circumference [OFC] 33 cm, on the 0.4^th^ centile), with a birth weight of 2880 Kg (10^th^ centile), a length of 49 cm (10^th^ centile), and an APGAR score of 9/10.

**Fig. 1:**
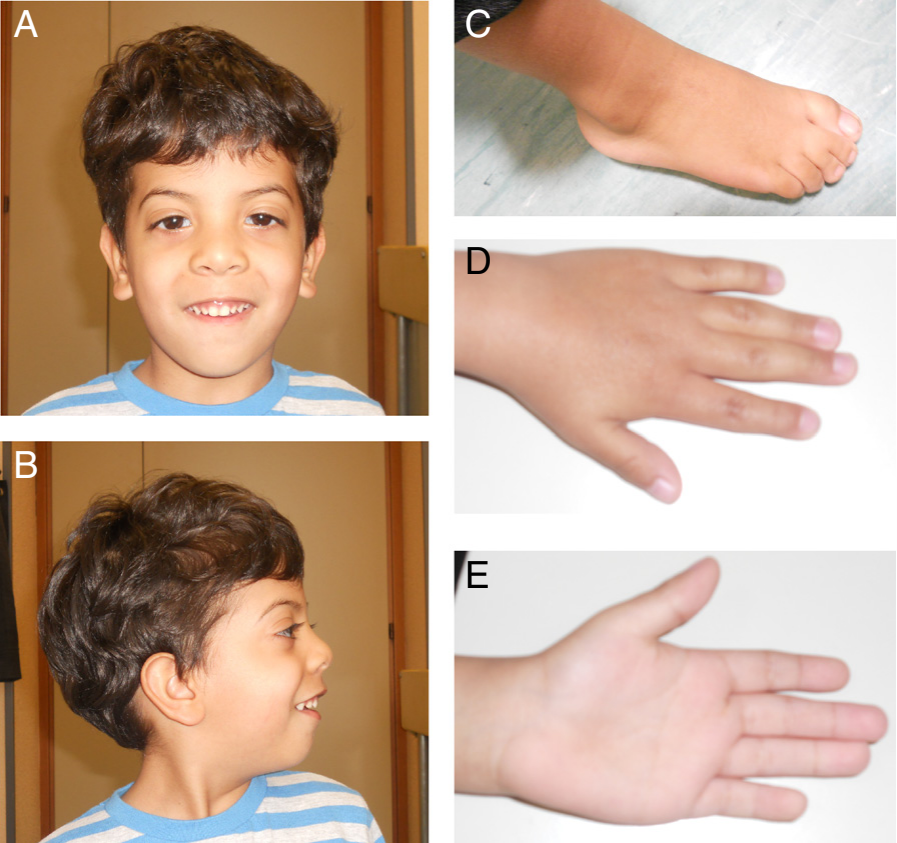
The patient's facial appearance (**a**, **b**) and the foot showing the 2,3 toe syndactyly (**c**), and the hands (**d**, **e**)

Postnatally, he demonstrated growth restriction with height and weight consistently on or just below the 0.4^th^ centile, and a head circumference growing well below the 0.4^th^ centile. He was noted to be slightly dysmorphic with bilateral ptosis, epicanthal folds, hypertelorism, a broad nasal bridge with anteverted nares, and posteriorly rotated ears. The hand and foot examination showed proximally placed thumbs, bilateral single hand creases, and partial bilateral 2–3 toe syndactyly (Fig. 1).

At 19 months of age, neuropsychiatric assessment revealed global hypotonia and borderline psychomotor development with a Griffith’s scale value of 93. At this stage, a number of investigations were carried out including cranial ultrasound, which was normal; electrocardiography, which was normal; and echocardiogram, which demonstrated a patent foramen ovale, then spontaneously closed; pelvic and abdominal ultrasound scan, which revealed bilateral pelvic ectasia more marked on the left consistent with bifid pelvis on this side; spine and hip X-ray, which were normal; and a metabolic screen, including urinary mucopolysaccharides, urinary oligosaccharides, and urinary organic acids, which were all normal. First-line genetic analyses were carried out: chromosome analysis by karyotype and array-CGH were normal. Given the dysmorphic features, the short stature and the cardiac defect, Noonan Syndrome was suspected. Molecular analysis of *PTPN11* and *SOS1* did not reveal any pathogenic mutation.

At the age of four years, neuropsychiatric assessment confirmed mild psychomotor delay, with an intelligence quotient (IQ) of 69 assessed by the Wechsler Preschool and Primary Scale. Growth parameters were as follows: weight 14 kg (3^rd^ centile), height 103 cm (10-25^th^ centile), and OFC 48 cm (<3^rd^ centile). At this stage, SLOS was suspected, and the molecular analysis of the *DHCR7* revealed the presence of two mutations: c.521 T > C (p.Phe174Ser), inherited from the mother and c.724C > T (p.Arg242Cys), inherited from the father, confirming the diagnosis.

With this report, we described a patient with a mild form of SLOS, who presented with subtle structural anomalies of the genitourinary tract associated with mild dysmorphic features and mild ID. The patient presented in this study demonstrated some of the typical SLOS facial dysmorphisms, microcephaly and growth failure. Despite being typical, these features are non-specific and are also present in other syndromic genetic conditions . Indeed, he was tested in the first place for Noonan syndrome, which was not confirmed. By and large, there were no clinical features that are pathognomonic for SLOS. Moreover, establishing a diagnosis can be complicated by the broad and variable phenotypic spectrum. The case here presented with bilateral syndactyly of the second and third toe, confirming the importance of this feature in establishing the diagnosis of SLOS. The patient was found to have bilateral ectasia of the pelvis and bifid pelvis on one side. Genitourinary malformations generally present in the most severe forms of the disorder with kidney agenesis, hypoplasia, ectopia [12] or with ambiguous genitalia, especially female-appearing genitalia or severe hypospadia [13]. Our case presented with an isolated dilatation of the pelvis,, highlighting the importance of subtle anomalies of the genitourinary tract in guiding diagnosis in the mild forms of SLOS.

Our case had two mutations in the *DHCR7* gene: p.Arg242Cys and p.Phe174Ser. Whilst the p.Arg242Cys mutation has been repeatedly reported in patients affected by SLOS [14, 15], the p.Phe174Ser has only been reported in one Portuguese patient [11]. The evidence of pathogenicity of this mutation is restricted to this report only, making it difficult to interpret this variant in a clinical setting.

**Fig. 2:**
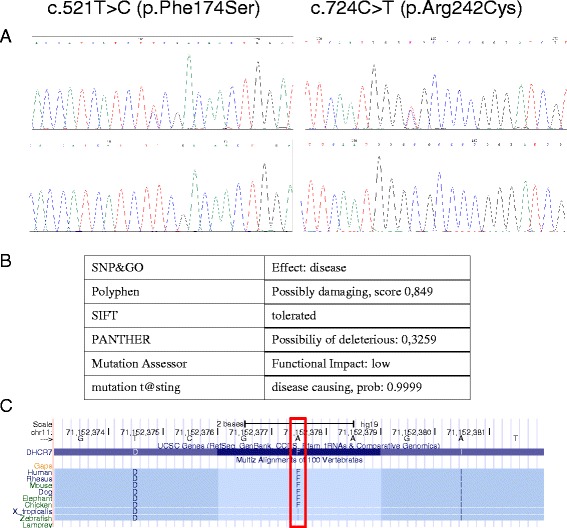
**a** Chromatograms showing the c.521 T > C (p.Phe174Ser), and the c.724C > T (p.Arg242Cys) mutations. **b** In silico programs showing the predicted effect of the p.Phe174Ser mutation on the DHCR7. SNP&GO, http://snps-and-go.biocomp.unibo.it/snps-and-go/; Polyphen, http://genetics.bwh.harvard.edu/pph2/; SIFT, http://sift.jcvi.org/; PANTHER, http://www.pantherdb.org/tools/csnpScoreForm.jsp; Mutation Assessor, http://mutationassessor.org/; Mutation t@sting, http://www.mutationtaster.org/ **c** Amino acid conservation between different species at the position 174 of the DHCR7

With this report, we provided further evidence of pathogenicity of this variant: (1) we describe this variant in association with another known pathogenic variant in a patient with SLOS; (2) the affected residue is phylogenetically highly conserved in human, dog, mouse, and rhesus monkey; (3) the Grantham difference is 154.81 and (4) the affected residue does not tolerate the phenylalanine to serine missense mutation according to *in silico* prediction programs such as SNP&GO, Polyphen, SIFT, PANTHER, Mutation Assessor and Mutation t@sting (Fig. 2).

Although genotype–phenotype correlation for SLOS is complicated by the broad mutational spectrum found in this disorder, most patients with very mild clinical and biochemical phenotypes are compound heterozygotes for two unique or uncommon missense mutations [13]. Recent studies suggested that those with one or two mutations in loop 1–2 or one mutation in the N-terminus usually have milder phenotypes, while the most severe phenotypes are observed in individuals with two null mutations or with two mutations in loop 8–9. The p.Phe174Ser and the p.Arg242Cys mutations are in the TM3 and TM5 domains, respectively. A patient who was compound heterozygous for two mutations in loop 3–4 of the TM5 domain has been previously reported with a mild form of SLOS [15], confirming the observation that carrying two mutations located near these domains causes a less severe phenotype. The patient presented in this study and the previously reported Portuguese patient carrying the p.Phe174Ser mutation are both affected by a form of SLOS on the mild end of the spectrum. Table 1 summarizes the differences between our case and the one with the p.Phe174Ser mutation that was previously reported. On the basis of these observations, it is possible to assume that the p.Phe174Ser variant is associated with mild forms of SLOS.

**Table 1:** Clinical features of patients carrying the p.Phe174Ser variant

	Our report	Cardoso et al., 2005 [11]
	c.[521 T > C]; [724C > T]	c.[IVS8-1G > C]; [521 T > C]
IQ	69	Normal
Behaviour	Normal behaviour	Behavioural problems
OFC	Microcephaly	Microcephaly
Brain	Normal	Normal
Mouth	Normal	Normal
Acral	Bilateral 2–3 toe syndactily	2-3 toe syndactily
Eye	Ptosis, epicanthal folds	Ptosis, epicanthal folds
Heart	Patent foramen ovale	Intraventricular communication
Kidney	Bifid pelvis	Normal
Liver	Normal	Information not available
Lung	Normal	Normal
Bowel	Normal	Feeding disorder
Genitalia	Normal	Information not available

*IQ* intellectual quotient, *OFC* occipitofrontal circumference

## Conclusions

We report a patient with a mild case of SLOS who presented with microcephaly, mild ID, and isolated pelvis ectasia. This highlights the importance of recognizing subtle anomalies of the genitourinary system associated with mild dysmorphic features and mild ID in establishing the diagnosis of mild forms of SLOS. This study offers further clinical significance to the p.Phe174Ser variant and provides evidence for its association with mild forms of SLOS. This finding further establishes a genotype–phenotype correlation for SLOS, which will facilitate counselling for this disorder and the prediction of therapeutic responses.

## Consent

Written informed consent was obtained from the patient’s parents for publication of this case report and any accompanying images. A copy of the written consent is available for review by the editor of this journal.

## Abbreviations

ID: intellectual disability
DHCR7: 7-dehydrocholesterol reductase
IQ: intelligence quotient
OFC: occipitofrontal circumference
SLOS: Smith Lemli Opitz syndrome

## References

[1] Fitzky BU, Witsch-Baumgartner M, Erdel M, Lee JN, Paik YK, Glossmann H, Utermann G, Moebius FF (1998). Mutations in the Delta7-sterol reductase gene in patients with the Smith-Lemli-Opitz syndrome. Proc Natl Acad Sci U S A.

[2] Waterham HR, Wijburg FA, Hennekam RC, Vreken P, Poll-The BT, Dorland L, Duran M, Jira PE, Smeitink JA, Wevers RA, Wanders RJ (1998). Smith-Lemli-Opitz syndrome is caused by mutations in the 7-dehydrocholesterol reductase gene. Am J Hum Genet.

[3] Wassif CA, Maslen C, Kachilele-Linjewile S, Lin D, Linck LM, Connor WE, Steiner RD, Porter FD (1998). Mutations in the human sterol delta7-reductase gene at 11q12-13 cause Smith-Lemli-Opitz syndrome. Am J Hum Genet.

[4] Nowaczyk MJM, Pagon RA, Adam MP, Ardinger HH (1998). Smith-Lemli-Opitz Syndrome. GeneReviews® [Internet].

[5] Bzduch V, Beholova D, Skoduva J (2000). Incidence of Smith-Lemli-Opitz syndrome in Slovakia. Am J Med Genet.

[6] Battaile KP, Battaile BC, Merkens LS, Maslen CL, Steiner RD (2001). Carrier frequency of the common mutation IVS8-1G > C in DHCR7 and estimate of the expected incidence of Smith-Lemli-Opitz syndrome. Mol Genet Metab.

[7] Nowaczyk MJ, Irons M (2012). Smith-Lemli-Opitz syndrome: phenotype, natural history, and epidemiology. Am J Med Genet C Semin Med Genet.

[8] Nowaczyk MJ, Whelan DT, Heshka TW, Hill RE (1999). Smith-Lemli-Opitz syndrome: a treatable inherited error of metabolism causing mental retardation. CMAJ.

[9] Tint GS, Irons M, Elias ER, Batta AK, Frieden R, Chen TS, Salen G (1994). Defective cholesterol biosynthesis associated with the Smith-Lemli-Opitz syndrome. N Engl J Med.

[10] Waterham HR, Hennekam RC (2012). Mutational spectrum of Smith-Lemli-Opitz syndrome. Am J Med Genet C Semin Med Genet.

[11] Cardoso ML, Balreira A, Martins E, Nunes L, Cabral A, Marques M, Lima MR, Marques JS, Medeira A, Cordeiro I, Pedro S, Mota MC, Dionisi-Vici C, Santorelli FM, Jakobs C, Clayton PT, Vilarinho L (2005). Molecular studies in Portuguese patients with Smith-Lemli-Opitz syndrome and report of three new mutations in DHCR7. Mol Genet Metab.

[12] Nowaczyk MJ, Eng B, Waye JS, Farrell SA, Sirkin WL (2003). Fetus with renal agenesis and Smith-Lemli-Opitz syndrome. Am J Med Genet A.

[13] Kelley RI, Hennekam RCJ (2000). The Smith-Lemli-Opitz syndrome. Med Genet.

[14] Neklason DW, Andrews KM, Kelley RI, Metherall JE (1999). Biochemical variants of Smith-Lemli-Opitz syndrome. Am J Med Genet.

[15] Krakowiak PA, Nwokoro NA, Wassif CA, Battaile KP, Nowaczyk MJ, Connor WE, Maslen C, Steiner RD, Porter FD (2000). Mutation analysis and description of sixteen RSH/Smith-Lemli-Opitz syndrome patients: polymerase chain reaction-based assays to simplify genotyping. Am J Med Genet.

